# Lifestyle and Quality of Life of Women Diagnosed with Hypothyroidism in the Context of Non-Alcoholic Fatty Liver

**DOI:** 10.3390/metabo13121174

**Published:** 2023-11-26

**Authors:** Barbara Janota, Brygida Adamek, Elżbieta Szczepańska, Krzysztof Biernacki, Ewa Janczewska

**Affiliations:** 1 Department of Basic Medical Sciences, Faculty of Public Health in Bytom, Medical University of Silesia in Katowice, 41-902 Bytom, Poland; 2 Department of Human Nutrition, Department of Dietetics, Faculty of Public Health in Bytom, Medical University of Silesia in Katowice, 41-808 Zabrze, Poland; 3 Department of Medical and Molecular Biology, Faculty of Medical Sciences in Zabrze, Medical University of Silesia in Katowice, Jordana 19, 41-808 Zabrze, Poland

**Keywords:** lifestyle, quality of life, hypothyroidism, non-alcoholic fatty liver

## Abstract

Interconnections between hypothyroidism and metabolic disturbances manifesting in the liver and body composition have not yet been comprehensively analyzed in the context of lifestyle. This study aimed to assess the selected lifestyle factors and quality of life in the context of the development of NAFL (non-alcoholic fatty liver) in women diagnosed with hypothyroidism. This study included 134 women categorized into three groups: with hypothyroidism and NAFL, with only hypothyroidism, and with only NAFL. We compared the groups concerning the KomPAN and WHOQOL-BREF questionnaires, anthropometric measurements, body composition parameters, and the stage of liver steatosis. The individuals with NAFL most frequently consumed lard, fried dishes, processed meats, red meat, sweets, and sweetened beverages. The individuals with hypothyroidism without coexisting NAFL exhibited the highest satisfaction with health. The NAFL group had the highest average body fat percentage. Selected lifestyle aspects influenced the development of NAFL in women diagnosed with hypothyroidism. Women’s overall quality of life did not vary depending on the coexisting medical conditions. Preventive programs should promote the following: the regular consumption of meals, the appropriate energy supply, physical activity, mental health support, and striving for proper body composition parameters.

## 1. Introduction

Non-alcoholic fatty liver (NAFL) resulting from metabolic disorders has become a global concern, prompting investigations into its causes. Coexisting hormonal imbalances and unhealthy lifestyles, including improper dietary behaviors [1], are among the potential predisposing factors for NAFL.

A special focus should be placed on thyroid hormones and all the liver–endocrine relationships that regulate hepatic metabolism to maintain metabolic homeostasis [2]. They control the oxidation of fatty acids, their accumulation, and the de novo production of carbohydrates and influence hepatic insulin sensitivity [2,3]. In patients with hypothyroidism, metabolic processes in the liver are disrupted, which can lead to hepatic steatosis [4]. Thyroid hormones significantly affect body composition, and hypothyroidism manifests as unfavorable changes. Thyroid hormone deficiency results in a decrease in the basal metabolic rate and an increase in fat levels, including visceral fat [5,6,7]. Increased visceral fat levels have also been observed in individuals with fatty liver [8]. The biochemical biomarkers often used in the group of patients with NAFL and hypothyroidism are, among others, glucose, lipoprotein, and thyrotropin concentration [4]. There are also imaging techniques, e.g., the assessment of the accumulation of fatty compounds within the liver parenchyma and the measurement of liver stiffness in hypothyroid groups of patients [4].

Lifestyle can influence the accumulation of fatty compounds in the liver and the presence of abnormal body composition parameters [9,10]. Preventive measures against developing NAFL, metabolic disorders, and body composition irregularities include engaging in regular physical activity and obtaining optimal sleep [11,12,13,14,15]. Dietary habits aimed at preventing the aforementioned abnormalities include adopting an anti-inflammatory diet rich in dietary fiber, plant-based products, and unsaturated fats while eliminating processed foods, fructose-rich products, and those that lead to a caloric surplus [16,17]. The Mediterranean diet fits this dietary model and is recommended for individuals with hypothyroidism [3,18]. This diet can have a beneficial effect in preventing metabolic disorders that may develop during the underlying condition [3,19,20,21].

The observed interconnections between hypothyroidism and metabolic disturbances manifesting in the liver and body composition have not yet been comprehensively analyzed in the context of lifestyle. Furthermore, the quality of life of individuals with coexisting NAFL and hypothyroidism, which diminishes when analyzed separately, has not been assessed [22,23,24,25].

This study aimed to assess the selected lifestyle factors in the context of the development of NAFL in women diagnosed with hypothyroidism. In addition, this study evaluated the quality of life and body composition of patients in the context of the occurrence of NAFL.

## 2. Materials and Methods

### 2.1. Study Group

This study was conducted between November 2022 and January 2023 at medical centers in Upper Silesia, Poland. In total, 134 women voluntarily participated in this study after providing informed consent. Of them, 106 were diagnosed with and underwent treatment for hypothyroidism, with regular endocrinological consultations. The remaining 28 women were diagnosed with NAFL through liver elastography. The inclusion criteria were as follows: female sex and age ≥ 18 years with a diagnosis of hypothyroidism or fatty liver disease. The exclusion criteria were as follows: male sex, age < 18 years, pregnancy, the presence of other endocrinological conditions, and the requirement for therapeutic or alternative dietary regimens.

The study participants were categorized into the following three groups in accordance with the medical diagnosis: group diagnosed with hypothyroidism and NAFL (78 individuals: H NAFL), group with only hypothyroidism (28 individuals: H), and group with only NAFL (28 individuals: NAFL).

### 2.2. Methodology

This study was conducted in three stages. In the first stage, the recruited patients were requested to complete standardized questionnaires: the KomPAN, a questionnaire for assessing dietary habits and attitudes, and the World Health Organization Quality of Life Brief Version, a questionnaire for evaluating quality of life [26,27]. In the second stage, anthropometric measurements were conducted, including waist circumference (cm) and hip circumference (cm), and body composition analysis was performed using the bioelectrical impedance method with a medical-grade InBody770 device in compliance with the International Organization for Standardization (ISO) 9001:2015 and ISO 13485:2016 standards. The following parameters were analyzed: skeletal muscle mass (SMM) (kg), percentage of body fat (%), extracellular water (ECW) index, visceral fat area (VFA) (cm^2^), phase angle (*), fat-free mass index (FFMI) (kg/m^2^), and fat mass index (FMI) (kg/m^2^). The final stage of this study involved assessing the stage of liver steatosis using a noninvasive method to evaluate the accumulation of fatty compounds within the liver parenchyma, known as the controlled attenuation parameter (CAP), and measuring the liver stiffness (LSM) using the FibroScan^®^ 502 Touch device. The examination was performed by individuals certified in performing liver elastography assessments and possessing a Hepatology Certification awarded by the Polish Hepatology Society [28,29].

### 2.3. Data Analysis

Statistical analysis was conducted using R version 4.3.1 with the ‘stats’ package in RStudio version 2023.06.1 Build 524 (PBC, Boston, MA, USA) [30]. We compared the study groups concerning the KomPAN and WHOQOL-BREF questionnaires, anthropometric measurements, body composition parameters, and the stage of liver steatosis using the Kruskal–Wallis test. The data were presented as the mean (M) and standard deviation (SD).

We also assessed the relationships between the study parameters within the study groups using the Spearman rank correlation coefficient (r_S_). Heat maps for the WHOQOL-BREF and body composition parameters with Euclidean clusterization were constructed using the ‘gplots’ package in R [31].

#### 2.3.1. Data Interpretation: KomPAN Questionnaire

Each response variant from the questionnaire was assigned scores according to its category.

The sections on the frequency of consuming selected food products were scored as follows: never, 1 point; 1–2 times per month, 2 points; once a week, 3 points; several times a week, 4 points; once a day, 5 points; and several times a day, 6 points.

The sections regarding sleep on working days and weekends were scored as follows: ≤6 h/day, 1 point; >6 but <9 h/day, 2 points; and ≥9 h/day, 3 points. The sections on physical activity on working days and weekends were scored as follows: low, 1 point; moderate, 2 points; and high, 3 points. The sections on screen time during the day were scored as follows: <2 h, 1 point; from 2 to almost 4 h, 2 points; from 4 to almost 6 h, 3 points; from 6 to almost 8 h, 4 points; from 8 to almost 10 h, 5 points; and ≥10 h, 6 points.

The mean (M) and standard deviation (SD) are presented for each group. The analysis included assessing the differences in the mean responses between the groups using the Kruskal–Wallis test. The responses obtained in the groups correlated with the stage of liver steatosis according to the presented scale. The correlation coefficient was interpreted as follows: r_S_ = 0, no correlation; 0 <|r_S_|> 0.3, weak correlation; 0.3 ≤ |r_S_| > 0.5, moderate correlation; 0.5 ≤ |r_S_| > 0.7, significant correlation; 0.7 ≤ |r_S_| > 0.9, high correlation; 0.9 ≤ |r_S_| > 1, very high correlation; and r_S_ = 1, full correlation [32]. The results were considered statistically significant in each case at *p* < 0.05.

#### 2.3.2. Data Interpretation: World Health Organization Quality of Life Brief Version

All the questionnaire questions were scored from 1 to 5 according to the authors’ assumptions. The analysis and correlation of the results followed the same approach used for the KomPAN questionnaire.

#### 2.3.3. Data Interpretation: Anthropometric and Body Composition

The results obtained during the measurements were averaged for each group. The presentation of the results, analysis, and correlations followed the same approach as the questionnaire data.

## 3. Results

The study group consisted of women aged 19–83 years. Of the 106 recruited individuals with thyroid dysfunction, 78 were diagnosed with NAFL, indicating liver steatosis in 73.6% of individuals with thyroid dysfunction. The characteristics of the study group with respect to the diagnosis are presented in Table 1.

### 3.1. Dietary Behaviors and Lifestyle

The characteristics of the eating behaviors are presented in Table 2.

The highest mean number of meals consumed was observed in the H group. The H group had the highest percentage of regular meal and snack consumption declarations. The highest percentage of sweetening drinks with sugar or honey occurred in the H NAFL group.

The mean frequency of the consumption of food products is presented in Table 3.

The H NAFL group had the highest frequency of vegetable, fermented dairy products, white meat, lard, cottage cheese, and sugar-sweetened beverage consumption. The H group had the highest frequency of fruit, whole-grain bread, legumes, fish, egg, butter, fast food, and packaged juice consumption. The NAFL group exhibited the highest frequency of whole grains, freshly squeezed juice, fried food, red meat, processed meat, and sweets consumption.

The frequency of consumption of the presented food products did not significantly differ among the groups (*p* ≥ 0.05).

In the H group, with increasing liver stiffness, there was a statistically significant decrease in the frequency of fish consumption. In the H NAFL group, with an increase in liver stiffness, there was a statistically significant increase in cottage cheese consumption frequency.

**Figure 1:**
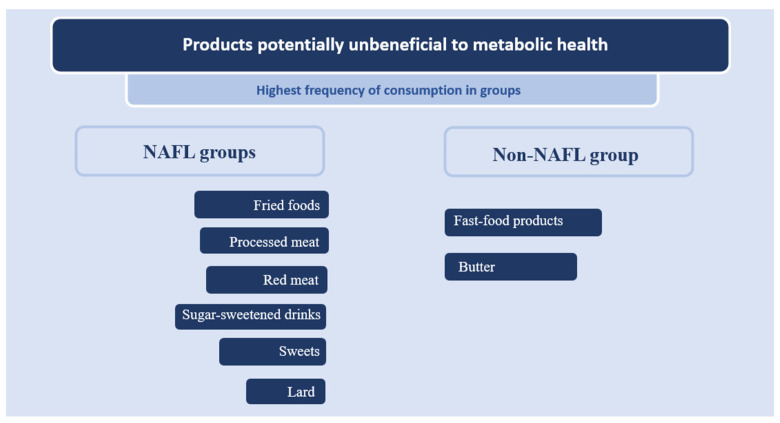
Most frequently consumed potentially metabolically unbeneficial products (based on the KomPAN questionnaire) are categorized into the non-alcoholic fatty liver (NAFL) and non-NAFL groups.

Figure 1 illustrates the most frequently consumed products potentially unbeneficial to metabolic health, categorized into groups with and without NAFL.

The individuals with NAFL exhibited the most frequent consumption of the following potentially metabolically detrimental products: lard, fried dishes, processed meats, red meat, sweets, and sweetened beverages. The individuals without NAFL (H only) showed the highest consumption of the following potentially metabolically unbeneficial products: butter and fast-food products.

The non-dietary aspects of lifestyle are presented in Table 4.

The NT group had the highest number of hours of sleep on weekends and the most favorable physical activity assessment. The NAFL group had the highest number of hours of sleep on weekdays and spent the most time using electronic devices. The responses regarding the non-dietary lifestyle factors did not significantly differ among the groups, and there were no significant correlations between the lifestyle factors and CAP (*p* ≥ 0.05). However, in the NAFL group, with an increase in liver stiffness, there was a statistically significant decrease in the level of assessment of activity during weekends (0 = 0.04; r_s_=−0.38).

### 3.2. Quality of Life

The quality of life of women is presented in Table 5.

The individuals with hypothyroidism without coexisting NAFL exhibited the highest satisfaction with health and self-satisfaction, had the best quality of sleep, had enough money compared to their needs, and experienced the most joy in life among the groups. In contrast, the prevalence of negative emotions was the lowest in this group.

In the NAFL group, an increase in the liver fat content was associated with decreased quality of life (*p* = 0.00; r_s_ = −0.6), physical appearance (*p* = 0.01; r_s_ = −0.47), and self-satisfaction (*p* = 0.04; r_s_ = −0.40). In the H NAFL group, an increase in liver fat content was associated with decreased support from friends (0 = 0.02; r_s_ = −0.26).

In the NAFL group, with an increase in liver stiffness, there was a statistically significant decrease in the quality of life (*p* = 0.04; r_s_ = −0.38) and satisfaction with health (*p* = 0.01; r_s_ = −0.50).

**Figure 2:**
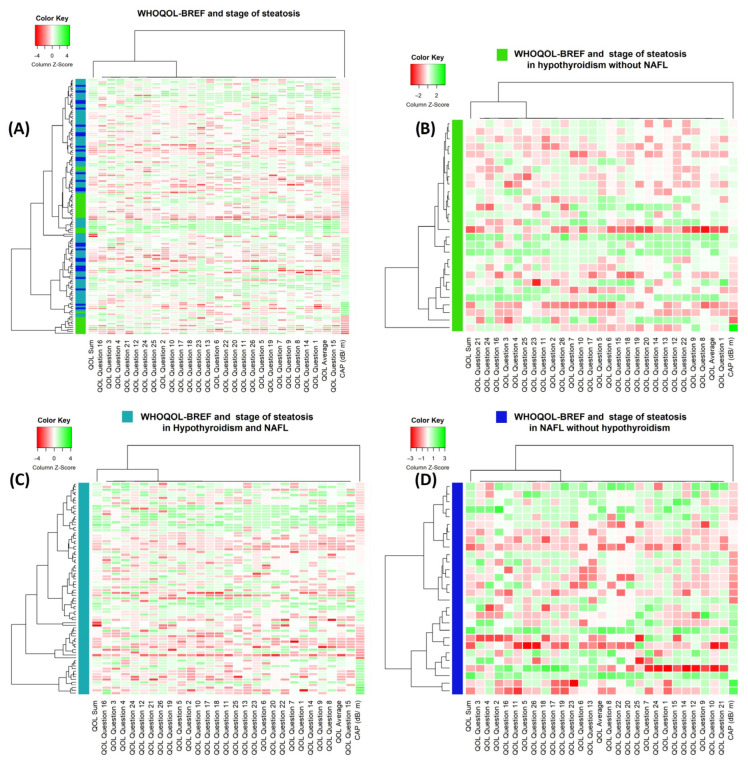
Heatmaps presenting clusterization results by cases (horizontally) and WHOQOL-BREF questionnaire answers, WHOQOL-BREF sum and average and stage of liver fat accumulation. (**A**) Heatmap and clusterization for total study cohort with case type (teal: H NAFL group, green: H group, and blue: NAFL group). (**B**) Heatmap and clusterization for H group. (**C**) Heatmap and clusterization for H NAFL group. (**D**) Heatmap and clusterization for NAHL group.

The clusterization resulted in limited patterns, with no significant grouping of the WHOQOL-BREF questionnaire answers in any significant way. The cases were also clustered in a manner that did not correspond to the group or fatty liver scale (Figure 2).

### 3.3. Body Composition Parameters

The results of the body composition analysis and anthropometric measurements in the respective groups are presented in Table 6.

The NAFL group had the highest body fat percentage, mean SMM, VFA, phase angle, lean body mass index, and FMI.

In the NAFL and H NAFL groups, with an increase in the CAP, there was a statistically significant increase in the SMM (*p* = 0.02; r_s_ = 0.44 and *p* = 0.04; r_s_ = 0.23), PBF (*p* = 0.00; r_s_ = 0.66 and *p* = 0.03; r_s_ = 0.25), VFA (*p* = 0.00; r_s_ = 0.7 and *p* = 0.02; r_s_ = 0.27), FFMI (*p* = 0.00; rs = 0.7 and *p* = 0.02; rs = 0.26), and FM (0.00; r_s_ = 0.72 and *p* = 0.01; r_s_ = 0.30). In the H group, with an increase in the CAP, there was a statistically significant decrease in the FFMI (*p* = 0.01; r_s_ = −0.48).

In the NAFL group, with an increase in the liver stiffness, there was a statistically significant increase in the PBF (*p* = 0.00; r_s_ = 0.60), VFA (*p* = 0.22; r_s_ = 0.60), FFMI (*p* = 0.00; r_s_ = 0.55), and FM (*p* = 00; r_s_ = 0.61).

**Figure 3:**
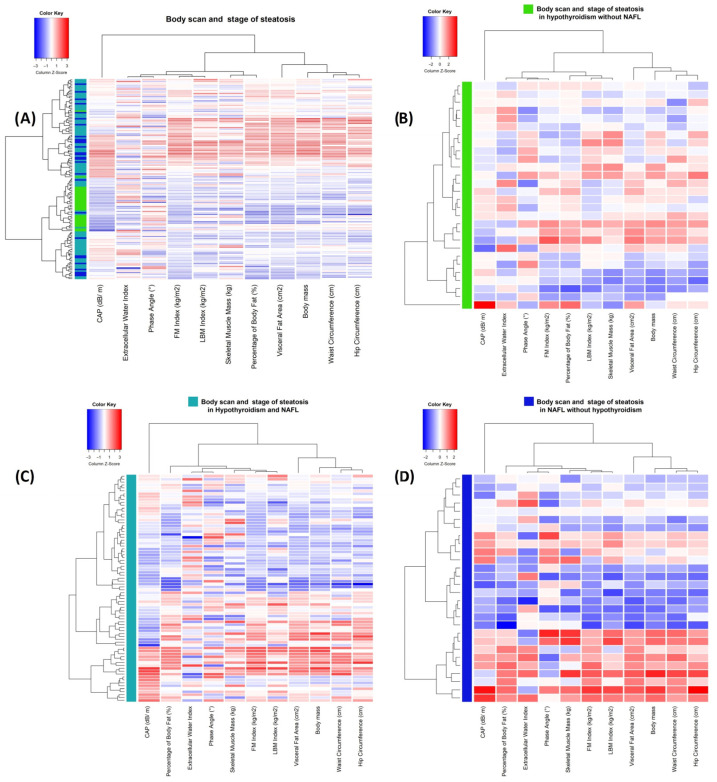
Heatmaps presenting clusterization results by cases (horizontally), body composition parameters, and stage of liver fat accumulation. (**A**) Heatmap and clusterization for total study cohort with case type (teal: H NAFL group, green: H group, and blue: NAFL group). (**B**) Heatmap and clusterization for H group. (**C**) Heatmap and clusterization for H NAFL group. (**D**) Heatmap and clusterization for NAHL group.

In all the groups (including the total study cohort), we can observe a grouping of the parameters into two groups. The grouping of the visceral fat area (cm^2^), waist circumference (cm), hip circumference (cm), and body mass is highly present in all the study groups. It is partially aligned with the steatosis, with lower values in the cluster typically corresponding to lower stages. No significant patient clusterization in the whole study cohort can be seen. However, the most prominent cluster of parameters has a higher representation of H cases for lower values of the parameters (Figure 3).

## 4. Discussion

Our study indicated the presence of NAFL in 73.6% of women treated for hypothyroidism. Not every publication has provided conclusive evidence of an increased probability of NAFL and its consequences in this patient group [33,34]. These divergent research results have prompted the exploration of the non-thyroidal factors that contribute to metabolic disturbances.

An analysis of dietary behaviors revealed the highest percentage of regular meal consumption in individuals with only hypothyroidism. This can prevent metabolic disorders in this group of women [35]. Alhussain et al. showed the positive effect of regular meal consumption on the plasma glucagon-like peptide 1 concentration, which increases the feeling of satiety and supports the control of food intake, affecting the better management of body weight and adipose tissue metabolism [35,36]. Moreover, there was a significant negative correlation between liver stiffness and the frequency of fish consumption in the only hypothyroidism group. It should be mentioned that according to Suárez et al., the Mediterranean diet as a source of, among others, fish products may be recommended in the dietary therapy of NAFL disease (NAFLD) [37]. Scientists also reported that this nutritional model reduces steatosis [38].

Among all the individuals with NAFL, compared with the individuals with hypothyroidism only, the highest consumption of red meat (3 vs. 2.5), processed meat (3.8 vs. 3.7), lard (1.9 vs. 1.5), fried foods (3.2 vs. 2.9), sweetened beverages (1.4 vs. 1.29), and sweets (3.4 vs. 3) was observed, which had the highest amounts of saturated fats and simple sugars. Donghia et al. also reported a higher incidence of liver fat accumulation among individuals of various ages who consumed processed and red meat more frequently than those without NAFL [39]. In a study from 2022 assessing the risk factors for, among other things, NAFLD in the British population, Guo et al. also indicated a significant positive correlation between the consumption of red meat and the development of NAFLD [40]. Iwanowska-Dajcman et al. further evaluated the risk of NAFLD occurrence based on the amount of meat consumed and showed that increased consumption of meat increased the risk of NAFLD [41]. However, Zhang et al. proved that replacing animal proteins with plant-based proteins reduced the risk [42].

Regarding the observation of lard and fried food consumption in the NAFL group, Das et al. suggested that it was mainly the type of fatty acids consumed, not their quantity, that influenced the development of fat accumulation in the liver [43]. Yabe et al. [44] demonstrated that consuming fried foods could lead to liver fibrosis. Saturated fatty acids (found in lard and margarine used for frying) have been identified as factors that lead to fat accumulation in the liver, which is in line with the results of our study [45]. Furthermore, Barroso et al. suggested incorporating fish oil supplementation rich in eicosapentaenoic acid, an unsaturated fatty acid, among patients with NAFLD to reduce the liver fat content [43,46].

An animal study showed that a high-carbohydrate diet was a greater risk factor for NAFL development than a high-fat diet [47]. In our study, the NAFL group exhibited a higher frequency of consumption of products rich in monosaccharides, which is consistent with the findings of Tseng et al., who reported a positive correlation between the consumption of monosaccharides in the form of sweetened beverages and NAFLD occurrence [48]. Based on a meta-analysis of 15 articles encompassing 15,149 individuals, Liu specified that fructose primarily caused liver fat accumulation [49].

Among the non-dietary factors that may affect liver health, our study found that individuals without NAFL rated their physical activity during working days (1.7 vs. 1.6) and weekends (1.8 vs. 1.6) most favorably and spent the least amount of time using electronic devices (2.7 vs. 2.6) compared with those in the NAFL groups. Moreover, there was a moderate negative correlation between liver stiffness and the assessment of physical activity on weekends. Montemayor et al. indicated that physical activity contributes to weight loss and could be considered a therapeutic element for NAFL [50]. Jafarikhah et al. suggested that resistance training could be beneficial in reducing liver fat [51]. However, Chen et al. noted that physical activity alone did not improve the liver condition in individuals with liver fat accumulation [52]. Notably, the patients without NAFL who slept the longest on weekends rated the quality of their sleep the best among all the groups. These results are consistent with those of Um et al., who demonstrated that poor sleep quality correlated with liver fat accumulation [53]. Ezpeleta et al. examined the effects of intermittent fasting and physical activity on sleep in individuals with NAFLD. Despite the favorable effects of these interventions on liver fat content and body mass, there was no improvement in sleep quality [54]. It seems that NAFL is a factor that may contribute to worsened sleep quality in patients with and without hypothyroidism.

Negative feelings, such as sadness, depression, and a depressive mood, occurred least frequently in the group without NAFL. Roa Dueñas et al. assessed the risk of depression occurrence depending on thyroid function among 9471 individuals [55]. They concluded that thyroid function could influence the occurrence of depressive episodes, but more often in hyperthyroidism [55]. For NAFLD and its metabolic counterpart, metabolic dysfunction-associated fatty liver disease, an independent association between metabolic liver disease and depression level has been proven [56]. These findings were presented in a study by Cai et al., who utilized data from 3263 participants in the National Health and Nutrition Examination Survey [56]. The data in the literature also indicates the need to identify eating disorders among individuals with NAFLD because the risk of personality disorders in this group is elevated, which can affect the control of food intake and exacerbate metabolic problems [57].

NAFL likely affects the level of health satisfaction, which is significantly lower among individuals with NAFL than those without. However, patients with concomitant diseases appear to adapt best to their overall situation, probably because of broader medical care. In their study of 244 Italians, Perez-Diaz-del-Campo et al. indicated that patients with NAFLD had a lower health-related quality of life, which deteriorated in cases of non-alcoholic liver fibrosis resulting from concurrent inflammation [58]. The data on the deteriorating health-related quality of life among individuals with non-alcoholic liver inflammation were also confirmed in the study by Younossi et al. [59]. Wang et al. also pointed out the association between NAFLD and the deteriorating health-related quality of life in the context of reduced physical mobility, underscoring the complexity of the problems faced by those with the condition [24]. Their study on quality of life suggested that interventions were necessary to reduce the VFA and increase muscle mass [24].

Muscle mass was the lowest in the groups with NAFL and NAFL with hypothyroidism. Researchers concur that the risks of sarcopenia and osteosarcopenia increase in patients with chronic liver disease [60,61]. Szlejf et al. demonstrated that the risk of muscle loss increased in hypothyroidism [62]. In contrast, the NAFL and NAFL with hypothyroidism groups exhibited the highest percentages of body fat and VFA. Notably, hypothyroidism alone is associated with an increase in the visceral fat index, as demonstrated by Pekgor et al. in their study of 68 patients [63].

The results of our study are consistent with those of previous studies, suggesting an association between lifestyle and metabolic disorders. However, the data regarding dietary behaviors are inconsistent. Publications from June to October 2023 suggest that it is not the type of food consumed but the overall dietary overconsumption and pro-inflammatory potential that leads to the development of NAFL [64,65].

One strength of this study is the concept of studying the coexistence of NAFL and hypothyroidism in the context of lifestyle, which aligns with the currently promoted holistic approach to disease prevention and therapy.

One of the limitations of this study is the inability to compare the results of our study with those of other authors due to a lack of available studies in the selected patient group. The lack of access to patient biochemical tests made correlating the liver fat stage with thyroid hormone concentrations impossible.

In the future, assessing the coexistence of NAFL and hypothyroidism would be valuable based on the amount of energy and nutrients supplied through the diet.

## 5. Conclusions

Selected lifestyle aspects, including dietary choices, influence the development of NAFL in women diagnosed with hypothyroidism. The overall quality of life of women does not vary depending on coexisting medical conditions, but individuals with hypothyroidism without NAFL exhibit the highest health satisfaction. NAFL is associated with having the least favorable fat parameters of body composition.

Preventive programs for the occurrence and progression of liver fat accumulation in high-risk groups should promote a diet with the following: regular meal consumption and the provision of the appropriate energy amount depending on individual needs.

It is recommended that physical activity is promoted to reduce weight and liver fat accumulation in patients with metabolic disorders and hypothyroidism.

## Figures and Tables

**Table 1:** Characteristics of the study group.

	Group H NAFL Hypothyroidism and NAFL		Group H Hypothyroidism without NAFL		Group NAFL NAFL without Hypothyroidism	
	Mean	SD	Mean	SD	Mean	SD
Age	59.5	13	56.6	16	56.8	12
BMI ^a^	28.4	6.6	24.4	2.9	30.9	7.1
WHR ^b^	0.87	0.08	0.83	0.08	0.95	0.09
WHtR ^c^	0.54	0.16	0.5	0.11	0.6	0.16
	Mean	N	Mean	N	Mean	N
Glucose (g/dL)	106	42	95.2	15	95.9	10
Total cholesterol (g/dL)	213.2	15	188.8	14	207.1	7
	N	%	N	%	N	%
Smoking (Yes)	9	11.5	5	17.9	3	10.7

^a^ Body Mass Index; ^b^ Waist–Hip Ratio; ^c^ Waist–Height Ratio. No significant differences (*p* > 0.05) were observed between the groups.

**Table 2:** Characteristics of eating behaviors.

Eating Behavior	H NAFL		H		NAFL	
	Mean	SD	Mean	SD	Mean	SD
Number of consumed meals	3.6	0.7	3.9	0.9	3.8	0.9
	N	%	N	%	N	%
Declaration of regular meal consumption	47	60.3	20	71.4	64.3	18
Declaration of snack consumption	67	86.9	26	92.9	22	78.6
Declaration of sweetening drinks (with sugar or honey)	27	34.6	7	25	6	21.4

**Table 3:** Mean frequency of consuming food products.

Product	Frequency of Consumption					
	H NAFL		H		NAFL	
	Mean	SD	Mean	SD	Mean	SD
Vegetables	4.77	0.95	4.75	1.27	4.46	0.74
Fruits	4.55	1.11	4.71	1.33	4.39	1.10
Whole-grain bread	3.31	1.78	3.48	1.78	3.43	1.77
Legume seeds	2.32	0.89	2.46	0.96	2.18	0.72
Whole grains	3.10	1.25	3.04	1.02	3.11	1.47
Fish	2.76	0.81	2.86	0.85	2.64	0.83
Fermented dairy products	3.53	1.26	3.44	1.37	3.46	1.07
White meat	3.54	0.77	3.29	1.15	3.43	0.84
Eggs	3.50	0.83	3.85	0.86	3.39	0.92
Freshly squeezed juices	2.17	1.29	2.18	1.09	2.21	1.10
Lard	2.13	1.53	1.52	0.85	1.71	1.01
Butter	4.12	1.65	4.41	1.76	4.32	1.36
Cottage cheese	3.29	1.23	2.89	0.93	3.04	1.23
Fried foods	3.08	1.21	2.89	1.31	3.29	1.18
Processed meat	3.63	1.23	3.74	1.51	3.96	0.79
Red meat	2.94	1.01	2.54	1.10	2.96	1.07
Fast food	1.63	0.79	1.71	0.60	1.57	0.74
Sweets	3.31	1.46	2.96	1.37	3.54	1.53
Sugar-sweetened drinks	1.49	1.04	1.29	0.53	1.29	0.46
Packaged juices	1.55	1.03	1.64	0.87	1.32	0.67

**Table 4:** Average responses in non-dietary factors of lifestyle.

Lifestyle Factor	H NAFL		H		NAFL	
	Mean	SD	Mean	SD	Mean	SD
Number of hours of sleep on weekdays	1.67	0.53	1.70	0.61	1.71	0.53
Number of hours of sleep on weekends	1.87	0.53	1.89	0.58	1.79	0.57
Assessment of activity during work or school days	1.62	0.69	1.74	0.66	1.57	0.50
Assessment of activity on weekends	1.67	0.66	1.78	0.70	1.57	0.57
Number of hours spent using electronic devices	2.63	1.42	2.56	1.25	2.75	1.55

**Table 5:** Quality of life.

Selected Element	H NAFL	H	NAFL
	Mean	Mean	Mean
Quality of life	3.75	3.70	3.50
Satisfaction with health *	3.30	3.36	2.89
Amount of joy in life	3.72	3.75	3.54
Concentration of attention	3.89	3.64	3.54
Level of energy	3.57	3.54	3.29
Physical appearance	3.76	3.61	3.50
Adaptability to circumstances *	3.87	3.54	3.50
Money compared to the needs	3.45	3.86	3.64
Quality of sleep	3.10	3.25	2.93
Self-satisfaction	3.67	3.89	3.57
Support of friends	4.14	3.93	4.04
Negative emotions (sadness, melancholy, depression)	3.5	3.4	3.6
Average: World Health Organization Quality of Life Brief Version	3.70	3.69	3.57

* Satisfaction with health differs significantly between the H and NAFL groups (*p* = 0.03) and between the H NAFL and H groups (*p* = 0.03). The level to which patients adapt to their current situation significantly differs between the H NAFL and H (*p* = 0.04) groups and the H NAFL and NAFL groups (*p* = 0.04).

**Table 6:** Average results of body composition analysis and anthropometric measurement results.

Body Composition Parameters	H NAFL		H		NAFL	
	Mean	SD	Mean	SD	Mean	SD
SMM (skeletal muscle mass) (kg)	26.1	3.6	23.7	37	26.3	4.7
PBF (percentage of body fat) (%) *	38.7	7.9	32.8	5.6	40.4	8.5
Extracellular water index	0.4	0	0.4	0	0.4	0
VFA (visceral fat area) (cm^2^) *	154.7	57.0	106.4	37.8	165.3	67.3
Phase angle (°)	5	0.6	5	0.7	5.2	0.5
FFMI (free fat mass index) (kg/m^2^) *	17.7	1.7	16.5	1.4	18.1	2.1
FMI (fat mass index) (kg/m^2^) *	11.9	4.6	8.2	2.4	13.2	5.4
Waist circumference (cm) *	94.4	12.99	84.3	9.4	101	176
Hip circumference (cm) *	108.2	12.52	102	7.8	106	10.8

* Significant differences (*p* < 0.05) were observed between the H and NAFL groups.

## Data Availability

The data will be available by contacting the corresponding author. Data is not publicly available due to privacy.
